# The activation of p38MAPK and JNK pathways in bovine herpesvirus 1 infected MDBK cells

**DOI:** 10.1186/s13567-016-0377-2

**Published:** 2016-09-02

**Authors:** Liqian Zhu, Chen Yuan, Liyuan Huang, Xiuyan Ding, Jianye Wang, Dong Zhang, Guoqiang Zhu

**Affiliations:** 1 College of Veterinary Medicine and Jiangsu Co-innovation Center for Prevention and Control of Important Animal Infectious Diseases and Zoonoses, Yangzhou University, 48 Wenhui East Road, Yangzhou, 225009 China; 2 The Test Center of Yangzhou University, 48 Wenhui East Road, Yangzhou, 225009 China

## Abstract

We have shown previously that BHV-1 infection activates Erk1/2 signaling. Here, we show that BHV-1 provoked an early-stage transient and late-stage sustained activation of JNK, p38MAPK and c-Jun signaling in MDBK cells. C-Jun phosphorylation was dependent on JNK. These early events were partially due to the viral entry process. Unexpectedly, reactive oxygen species were not involved in the later activation phase. Interestingly, only activated JNK facilitated the viral multiplication identified through both chemical inhibitor and siRNA. Collectively, this study provides insight into our understanding of early stages of BHV-1 infection.

## Introduction

Bovine herpesvirus 1 (BHV-1), an enveloped virus belonging to the* alphaherpesvirus* subfamily, infects cattle of all ages and breeds worldwide. BHV-1-induced immune suppression initiates the secondary bacterial infection and leads to bovine respiratory disease complex, ultimately resulting in high mortality [1, 2]. The viral infection may also result in abortions, inflammation, conjunctivitis, and severe neonatal diseases. It costs the US cattle industry approximately 3 billion dollars annually [3].

Mitogen-activated protein kinases (MAPK), a family of serine/threonine protein kinases, are mainly divided into three family members including the extracellular signal-regulated kinase 1 and 2 (Erk1/2), c-Jun NH2-terminal kinase (JNK) and p38MAPK [4, 5]. They phosphorylate specific substrates at serine and/or threonine residues, and thereby transduce signals from the cell membrane to the nucleus in response to a wide range of stimuli, to participate in a diverse array of cellular programs including cell mitosis, proliferation, motility, metabolism, and other fundamental biological processes [6, 7]. Accumulated evidence indicates that MAPK pathways are involved in inflammatory response via activating the target genes of inflammatory mediators [8–10]. Moreover, inhibitors targeting p38MAPK and JNK pathways have been developed for anti-inflammatory therapeutics, and the data from preclinical treatments have validated their prominent anti-inflammatory effect [11].

Since the MAPK cascades broadly regulate cellular biology function, it is not surprising that they are involved in the pathological responses of hosts to viral infection. For example, MAPK pathways were implicated in inflammatory response by the infection of influenza virus and HSV-1 [12–15]. The employment of MAPK inhibitors emerges as an attractive strategy to reduce both viral load and the level of pro-inflammatory cytokines to definitely control viral infection. We know that BHV-1 infection activates MAPK/Erk1/2 signaling in MDBK cells [16]. However, little is known about the response of p38MAPK and JNK in BHV-1 infection.

The aim of this study was to determine whether BHV-1 infection could alter p38MAPK and JNK pathways in MDBK cells. We found that BHV-1 infection of MDBK cells indeed activated both p38MAPK and JNK pathways. However, only the JNK pathway was essential to viral replication. We also defined that c-Jun was exclusively activated by viral infection through JNK. Unexpectedly, BHV-1 infection-activated MAPK pathways was not through a reactive oxygen species (ROS)-dependent mechanism, though ROS is widely reported to be an activator of MAPK pathways during numerous virus infections, such as by HSV-1 [17, 18]. These studies partially address the importance of MAPK pathways in BHV-1 infection induced inflammatory response.

## Materials and methods

### Antibodies and reagents

Antibodies against phospho-JNK (Thr183/Tyr185), phospho-p38MAPK (Thr180/Tyr182), Phospho-p44/42 MAPK (Erk1/2) (Thr202/Tyr204), phospho-c-Jun (Ser73), JNK, p38MAPK, p44/42 MAPK (Erk1/2), c-Jun, and GAPDH, as well as HRP labeled secondary antibodies anti-mouse IgG or anti-rabbit IgG were purchased from Cell Signaling Technology (Beverly, MA, USA). *N*-Acetyl-l-cysteine (NAC) was bought from Sigma-Aldrich (St. Louis, MO, USA). U0126, SB203580, and SP600125 were purchased from Cell Signaling Technology. BHV-1 VP16 antibody is kindly provided by Prof. Vikram Misra at the University of Saskatchewan [19].

### Virus and cell cultures

MDBK cells (provided by Dr Leonard J. Bello, University of Pennsylvania) were maintained at 37 °C in 5% CO_2_ in DMEM (Gibco BRL) supplemented with 10% horse serum (HyClone Laboratories, Logan, UT, USA). BHV-1 Colorado1 strain (provided by Dr Leonard J. Bello, University of Pennsylvania) used for this study was propagated in MDBK cells. Aliquots of virus stocks were stored at −70 °C until use. The virus was titrated in MDBK cells with results expressed as TCID_50_ calculated using the Reed-Muench formula. The inactivation of BHV-1 by UV-irradiation was performed as previously described [16]. Effective inactivation of viruses was confirmed by virus titer assay in MDBK cells.

### siRNA knockdown

siRNA1–3 targeting JNK1, and siRNA4–6 targeting JNK2 as well as the control siRNA were purchased from Genepharma (Shanghai, China). SiRNA transfection was performed with transfection reagent siRNA-Mate (Genepharma) according to the manufacturer’s specifications. Efficiency of these siRNA were characterized by Western blotting.

### Inhibition of viral replication by chemical inhibitors

Confluent MDBK cells in 24-well plates were pretreated with the detected inhibitors at the indicated concentrations for 1 h at 37 °C, followed by BHV-1 infection at MOI of 1 for 1 h. After extensive washing with PBS, the cells were replaced with fresh medium DMEM (400 μL) with or without chemicals and cultured in a CO_2_ incubator at 37 °C. The virus yield was titrated by TCID_50_ assay.

### Western blot analysis

Monolayers of MDBK cells in 60-mm dishes were serum starved overnight, mock-infected or infected with BHV-1 (MOI = 10) at 37 °C for 0.5, 1, 2, 4, 8, 12 and 24 h. Cell lysates were prepared with lysis buffer (1% Triton X-100, 50 mM sodium chloride, 1 mM EDTA, 1 mM EGTA, 20 mM sodium fluoride, 20 mM sodium pyrophosphate, 1 mM phenylmethylsulfonyl fluoride, 0.5 g/mL leupeptin, 1 mM benzamidine, and 1 mM sodium orthovanadate in 20 mM Tris–HCl, pH 8.0) at the indicated time points. To analyze the effect of detected inhibitors on the designated signaling, MDBK cells were exposed to the corresponding chemicals through virus infection plus a pretreatment for 1 h before virus inoculation. To analyze the effect of siRNA on the designated signaling, MDBK cells were transfected with siRNA and incubated for 48 h, then infected with BHV-1 for the indicated time length at 37 °C.

Cell lysates were separated on 8 or 10% SDS–polyacrylamide gels and transferred to a polyvinylidene difluoride (PVDF) membrane (Bio-rad, CA, USA). After blocking with 5% nonfat milk in Tris-buffered saline (TBS) buffer containing 0.05% Tween 20 (TBST), the membrane was incubated with respective primary antibodies, and followed by HRP-conjugated secondary antibodies in the blocking reagent. After extensive washing with TBST, immune reactive bands were detected by film exposure after enhanced chemiluminescence (ECL) reaction (Millipore, USA).

## Results

**Figure 1:**
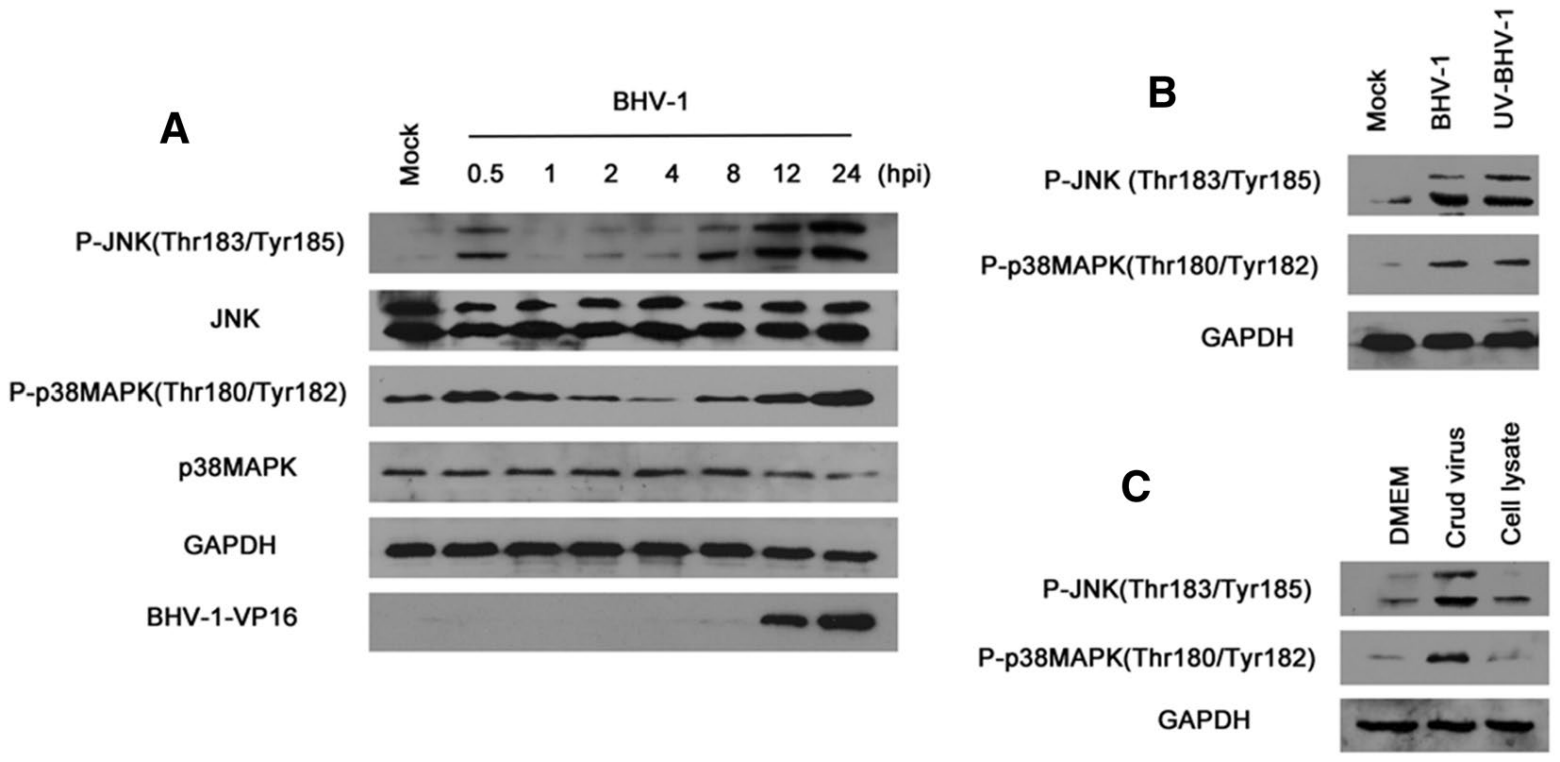
**Biphasic activation of both JNK and p38MAPK pathway in BHV-1 infected MDBK cells. A** Time course of altered phospho-JNK(Thr183/Tyr185) and phosphor-p38MAPK(Thr180/Tyr182) following BHV-1 infection. Growth arrested MDBK cells were mock infected or infected with BHV-1 at an MOI of 10. At the indicated time points, cells were lysed and subjected to Western blotting analysis using the indicated antibodies. **B** UV-irradiation inactivated virus also induced activation of both JNK and p38MAPK. MDBK cells were infected with BHV-1 (MOI = 10) or with equal amounts of UV-irradiation inactivated virus for 30 min. The cells were lysed for Western blotting. **C** Uninfected cell lysates could not stimulate p38MAPK and JNK signaling. To get uninfected cell lysates and crude virus, MDBK cells were mock infected or infected with BHV-1 (MOI = 1) without serum in the medium. At 48 hpi, the cell cultures were subjected to frozen-thawing and centrifugation at 15 000 rpm for 10 min. The supernatants were collected and used for the treatment of MDBK cells. At 0.5 h post-treatment, the cells were lysed for Western blotting analysis. Data are representative results of three independent experiments.

We previously reported that BHV-1 infection induces the activation of MAPK/Erk1/2 signaling in MDBK cells [16]. To identify whether JNK and p38MAPK signaling were involved in BHV-1 infection of MDBK cells, we first examined the kinetics of phosphorylated JNK and p38MAPK after BHV-1 virus infection using Western blotting. To see the burst effect of virus infection on the detected signaling pathways, serum starved MDBK cells were infected with BHV-1 at a high MOI of 10. As a result, the levels of both phospho-JNK (Thr183/Tyr185) and phospho-p38MAPK (Thr180/Tyr182) were dramatically elevated in a two-tiered manner following BHV-1 infection (Figure 1A). The first tier was transiently increased at the early stage of virus infection (Figure 1A, 0.5 hpi). The second wave was sustained at the later stage of infection (Figure 1A, 12 and 24 hpi). Obviously, the alteration of phosphorylated p38MAPK or JNK was neither due to overexpression of total p38MAPK (or JNK) nor to a high amount of protein loading (Figure 1A). Here, the lytic cycle viral protein VP16 of BHV-1 was detected to identify the kinetics of viral infection. Interestingly, the kinetics of the second tier of activated p38MAPK and JNK correlated with that of viral protein VP16 expression (Figure 1A). It is highly possible that the de novo viral protein expression and/or DNA replication account for the second tier of activated p38MAPK and JNK.

Considering that the early-transient activation of both p38MAPK and JNK appeared as early as 0.5 hpi, UV-irradiation inactivated virus could enter cells but not initiate subsequent genomic replication nor translation. We employed the UV-irradiation inactivated virus to explore the role of the viral entry process on these activations. UV-irradiation inactivated viruses were generated with infectious virus exposed to a 30 W UV light at a distance of 10 cm for 1 h. Complete inactivation of virus was confirmed with a virus titer assay (data not shown). MDBK cells were inoculated with the infectious virus (MOI = 10) or equal amounts of UV-inactivated virus in parallel for 0.5 h. As shown in Figure 1B, UV-irradiated BHV-1 induced similar levels of p38MAPK and JNK activation when compared to functional BHV-1, suggesting that an early step(s) of viral infection would be responsible for these events.

Though we generated the virus stocks without serum in the cell culture, both cell debris or growth factors were still present in the crude virus, which may have stimulated JNK and p38MAPK signaling. The supernatant from uninfected cell culture was obtained in parallel to the virus stock. MDBK cells were treated with either supernatant of uninfected cell culture or crude virus at 37 °C for 0.5 h, the phosphorylation of both JNK and p38MAPK was detected using Western blotting. As a result, the supernatant of uninfected cell culture could not induce apparent activation of both JNK and p38MAPK (Figure 1C). It ruled out the possibility that the activation of JNK and p38MAPK signaling by UV-inactivated virus was due to the cellular debris or the presence of growth factors in the medium.

**Figure 2:**
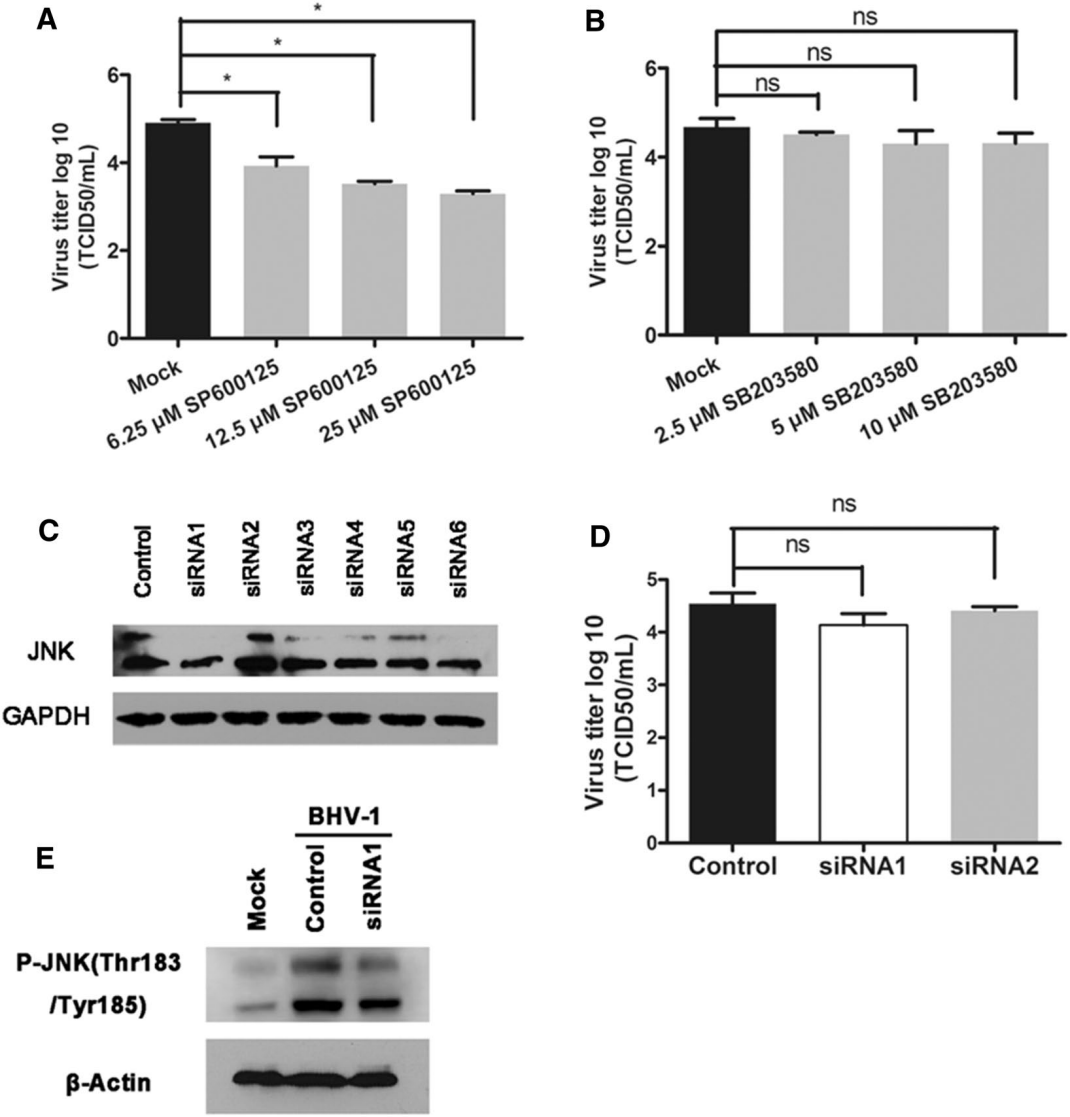
**The JNK but not p38MAPK signaling related to BHV-1 replication. A**, **B** JNK inhibitor inhibited BHV-1 replication. MDBK cells were first pretreated with inhibitor SP600125 **A** specific for JNK or inhibitor SB203580 **B** specific for p38MAPK at various concentrations for 1 h, and then infected with BHV-1 at an MOI of 1. At 24 hpi, virus yield was titrated using the TCID_50_ assay. **C** Screening siRNA target for JNK1 and JNK2. MDBK cells in 6-well plates were transfected with the indicated siRNA of 100 pM using transfection reagent siRNA-Mate (Genepharma) according to the specifications. At 48 h post transfection, the cells were lysed and subjected to Western blotting with antibody against JNK. **D** Knock down of the expression of JNK1 with siRNA1 moderately inhibited BHV-1 replication. MDBK cells in 24-well plates were transfected with siRNA1, siRNA3 and control siRNA of 20 pM using transfection reagent siRNA-Mate. At 48 h post transfection, the cells were infected with BHV-1 at MOI of 1 for 1 h, after extensive washing with PBS, fresh medium was replaced for further incubation of 24 h. Virus yield was tittered with TCID_50_. The data are from three independent experiments. Statistical analyses were performed using the Student’s t test (P < 0.05 vs. control). **E** Knockdown JNK1 by siRNA1 reduced JNK1 phosphorylation induced by BHV-1 infection. MDBK cells in 6-well plates were transfected with siRNA1 and control siRNA, respectively. At 48 h post-transfection, the cells were infected with BHV-1 at MOI of 10 for 0.5 h. The cell lysates were prepared for Western blotting analysis. Data are representative of two repeats.

To investigate the role of p38MAPK and JNK pathways in viral amplification, chemical inhibitors SP600125 and SB203580 specific for JNK and p38MAPK, were respectively employed to treat MDBK cells during the virus infection. Proper concentration for each inhibitor that showed no cytotoxicity to MDBK cells was selected based on the specifications and 3-(4,5-dimethyl-2-thiazolyl)-2,5-diphenyl-2H-tetrazolium bromide (MTT) assay (data not shown). The treatment with JNK specific inhibitor SP600125 at 6.25, 12.5 and 25 μM strongly reduced the virus production ranging from ~1 to ~1.6 logs (Figure 2A). Although p38MAPK was activated by BHV-1 infection, the p38MAPK specific inhibitor SB203580 showed no apparent effect on viral replication (Figure 2B). The data suggest that JNK pathways play an important role in viral amplification.

The JNK are encoded by three separate genes (JNK1, 2, and 3), which are spliced alternatively to create 10 JNK isoforms that are either 54 or 46 kDa in size [20]. In mammalians, JNK1 and JNK2 are widely expressed, whereas JNK3 expression is largely restricted to the brain [21, 22]. Chemical inhibitors might exert off-target effects. We used siRNA-mediated knock down to address the specific role of JNK in BHV-1 replication. MDBK cells were transiently transfected with siRNA1–3 targeting JNK1, and siRNA4–6 targeting JNK2 as well as the control siRNA (provided by Genepharma, Shanghai, China). Of these detected siRNA, only siRNA1 could apparently reduce the expression of JNK as determined by Western blotting (Figure 2C). Next, MDBK cells in 24-well plates were transfected with siRNA1, siRNA2 and control siRNA, respectively, and at 48 h post-transfection they were infected with BHV-1 (MOI = 1). The virus yield was determined at 24 hpi with TCID_50_ assay. As a result, comparing to either control siRNA or siRNA2, siRNA1 moderately decreased viral titer by ~0.5 log (Figure 2D). Although the inhibitory effect of siRNA1 was not as strong as the inhibitor SP600125 on viral replication, knockdown of JNK1 consistently decreased the virus infection. In addition, siRNA transfection reduced the level of phosphorylated JNK (Figure 2E), which corroborated the result that BHV-1 infection activated JNK, and knockdown of JNK1 reduced BHV-1 replication. Collectively, these results imply that JNK1 may be involved in BHV-1 replication.

**Figure 3:**
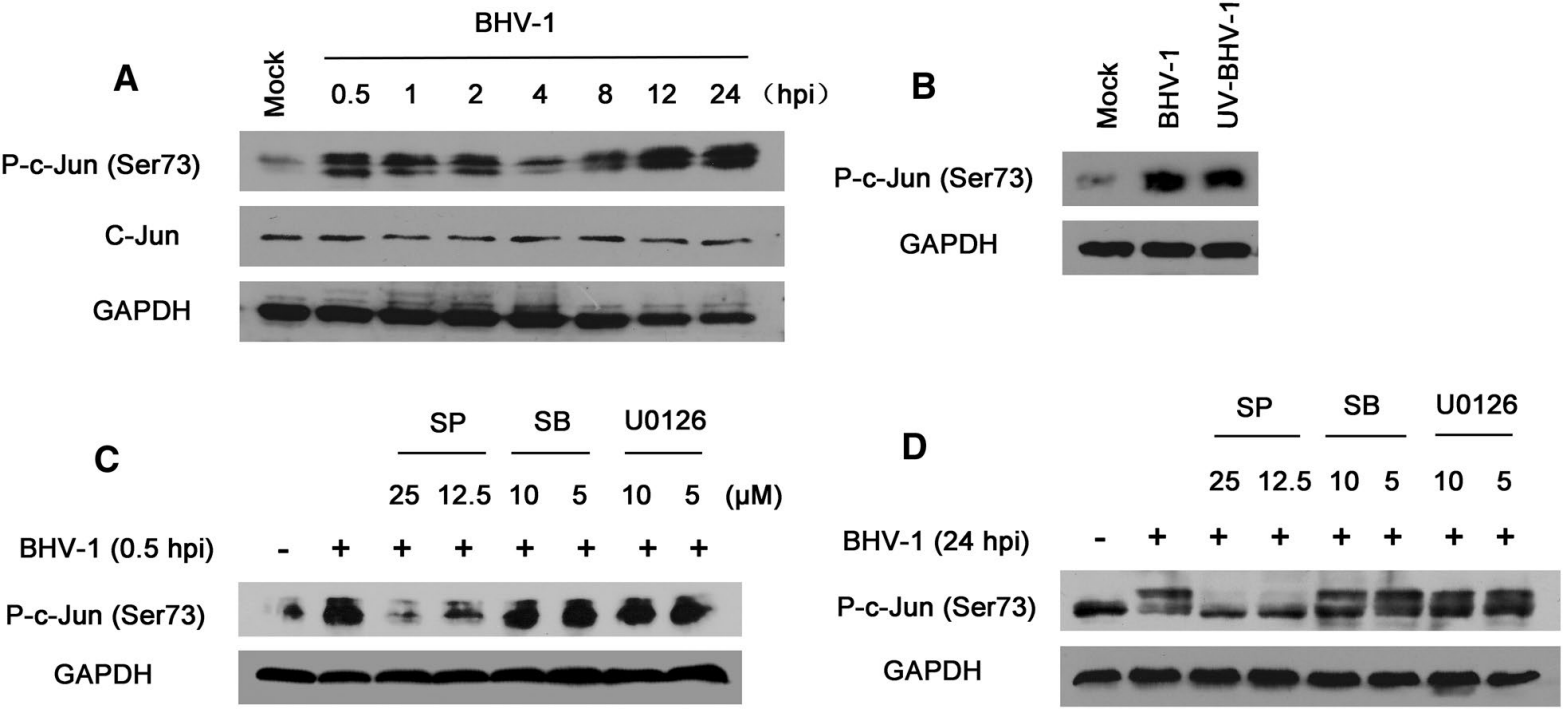
**The activation of c-Jun following BHV-1 infection depended on JNK signaling. A** Time course of altered phosphorylation of c-Jun at Ser73 following BHV-1 infection. Growth arrested MDBK cells were mock infected or infected with BHV-1 at an MOI of 10. At the indicated time points, the cells were lysed and subjected to Western blotting analysis using specific antibodies. **B** UV-irradiation inactivated virus still induced activation of c-Jun. MDBK cells were infected with BHV-1 (MOI = 10) or with equal amounts of UV-irradiation inactivated virus for 30 min. The cells were lysed for Western blotting. **C, D** C-Jun was activated by BHV-1 through JNK. MDBK cells were first pretreated with inhibitor SP600125, SB203580 and U0126 specific for JNK, p38MAPK and Erk1/2 signaling at the indicated concentrations for 1 h, and then infected with BHV-1 at an MOI of 10. At 0.5 and 24 hpi, the cells were respectively lysed for Western blotting to detect the phospho-c-Jun. Data are representative results of three independent experiments.

Upon activation, the phosphorylated JNK form dimers and enter the nucleus to activate target genes through its effects on c-Jun, ATF-2, and other transcription factors [23]. We therefore subsequently monitored the kinetics of activated c-Jun during BHV-1 infection through a Western blotting assay. The elevated levels of phosphorylated c-Jun (Ser73) were observed at both early and later stages of virus infection (Figure 3A). Exposure to UV-inactivated virus rendered an early event of c-Jun phosphorylation, as determined at 0.5 hpi at a level comparable to that obtained with functional virus (Figure 3B). It implies that BHV-1 infection activated c-Jun pathways, and viral early entry steps could be responsible for the early wave of activation.

Although c-Jun indeed is a canonical downstream target of JNK, accumulated data has shown that c-Jun can also be activated by other MAPK signaling axes such as by p38MAPK and Erk1/2. For example, JNK independent phosphorylation of c-Jun has been demonstrated in numerous cells stimulated with phorbol 12-myristate 13-acetate (PMA) [24], and c-Jun-dependent microglial inflammatory response following irradiation is mediated by Erk1/2 but not by JNK [25]. Here, we chemically inhibited JNK signaling with SP600125, p38MAPK signaling with SB203580 and Erk1/2 signaling with U0126, to elucidate whether there was a dialog between c-Jun and p38MAPK and Erk1/2. MDBK cells were pretreated with compounds at indicated concentrations for 1 h and then infected with BHV-1 (MOI = 10) in the presence of inhibitors. At 0.5 and 24 hpi, the cells were lysed and p-c-Jun was detected by Western blotting. Interestingly, BHV-1 induced phosphorylation of c-Jun was uniquely inhibited by JNK inhibitor SP600125 (Figures 3B and C). This indicates that JNK is the unique stimulator for BHV-1-activated c-Jun signaling. Similarly, it has been reported that treatment of HSV-1-infected Vero cells with SB203580 results in minor effects on c-Jun activation [26].

**Figure 4:**
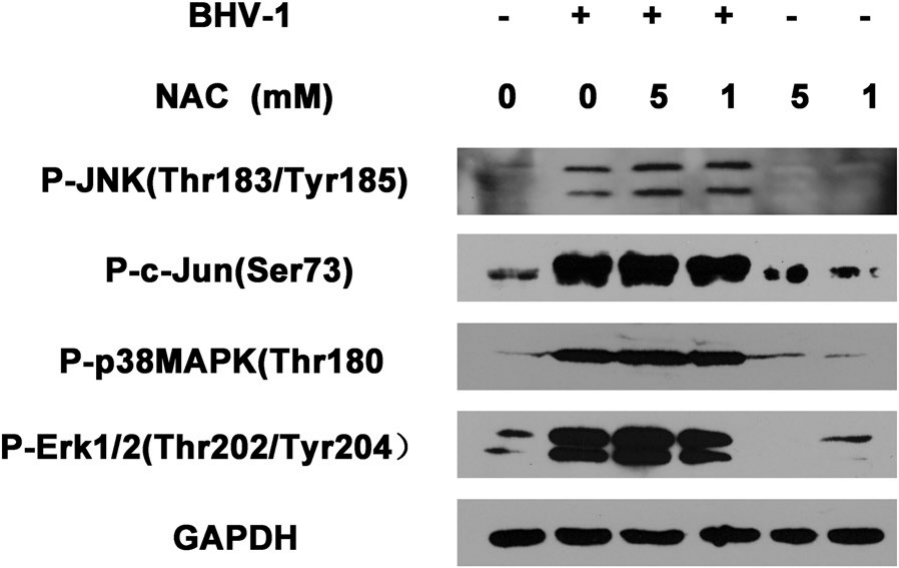
**The effect of ROS scavenger NAC on BHV-1 stimulated MAPK pathways.** MDBK cells were first mock pretreated with DMSO control or pretreated with NAC at the indicated concentrations for 1 h, then mock infected or infected with BHV-1 at an MOI of 10 along with NAC or DMSO control. At 24 hpi, cell lysates were prepared and phosphorylated forms of JNK, c-Jun, p38MAPK and Erk1/2 were detected using Western blotting. Data represent three independent experiments.

Reactive oxygen species are key signaling molecules that play an important role in the progression of inflammatory disorders [27]. Our recent data indicate that BHV-1 infection stimulates ROS production to facilitate viral infection and mediate mitochondrial dysfunction [28]. HSV-1 is genetically close to BHV-1, which stimulates MAPK signaling with a ROS-dependent manner [10]. Therefore, the relation between ROS production and MAPK activation induced by BHV-1 infection was investigated. MDBK cells were treated with ROS scavenger NAC [29] at a concentration of 5 and 1 mM, respectively, and infected with virus (MOI = 10). At 24 hpi the cells were lysed for Western blotting to detect the phosphorylation of p38MAPK, JNK, Erk1/2 and c-Jun. As a result, none of them were blocked by ROS scavenger NAC (Figure 4). This suggests that BHV-1 stimulated the three MAPK signaling, p38MAPK, Erk1/2 and JNK/c-Jun with a ROS-independent manner, which was distinct from that of the genetic closely related HSV-1.

## Discussion

The canonical MAPK pathways implicated in various inflammatory responses have been reported to be modified by diverse viruses to support productive replication, control cell proliferation, suppress cell apoptosis, or induce cytokine production and inflammation [10, 30, 31]. For example, the activation of p38MAPK and Erk1/2 is required for the nuclear export of viral ribonucleoprotein complexes [32, 33]. Here, we established that both p38MAPK and JNK signaling were activated in BHV-1-infected MDBK cells (Figure 1). Together with our previous data [16], we knew that in BHV-1 infected MDBK cells the three canonical MAPK signaling including p38 MAPK, JNK and Erk1/2 were activated. BHV-1 and HSV-1 belong to the *Alphaherpesvirinae* subfamily and share a number of biological properties. However, HSV-1 infection activates both p38 MAPK and JNK signaling, but reduces Erk1/2 signaling [14, 34, 35]. Obviously, these MAPK pathways were differentially manipulated by BHV-1 and HSV-1. It is reasonable that the discriminatory controlling of MAPK pathways would produce different effects on virus pathogenicity. In the future it would be interesting to study the mechanisms of the differential manipulation of MAPK pathways by these two viruses.

Since the UV-inactivated viral particles could enter host cells but not complete subsequent gene transcription, they could still activate these MAPK signaling at 0.5 hpi. Based on this data and our previous report [16], we suggest that the viral entry process may partially account for the enhanced phosphorylation of these three MAPK signaling. ROS are important inflammatory mediators, which are recognized as secondary messengers to activate a variety of cellular signaling pathways such as p38MAPK and Erk1/2 after HSV-1 infection of murine microglial cells [10]. We recently reported that BHV-1 infection increases ROS production, which contributes to viral replication [28]. Considering that BHV-1 and HSV-1 are genetically closely related, ROS is a putative component responsible for BHV-1 activated MAPK pathways. However, here we found that ROS was not accounted for BHV-1-stimulated phosphorylation of p38MAPK, Erk1/2 and JNK (Figure 4). So the activation of these MAPK pathways by BHV-1was not mediated by ROS.

JNK activation may exert viral-supportive or antiviral effect for diverse viruses. For example, JNK knockout mouse embryonic fibroblasts (MEF) were more susceptible to oncolytic vaccinia virus infection than wild-type MEF [36]. In contrast, JNK inhibitor SP600125 possesses a strong inhibitory effect on viral replication of either highly pathogenic avian virus strain A/FPV/Bratislava/79 (H7N7) or the pandemic swine-origin influenza virus A/Hamburg/4/09 (H1N1v) [37]. Here, we elucidated that only JNK was required to support BHV-1 replication, but p38MAPK could not (Figures 2A, B, D). Interestingly, both P38 MAPK and JNK pathways are important for HSV-1 gene expression and viral propagation [26, 34, 38]. So the MAPK pathways are discriminately controlled by BHV-1 and HSV-1 for viral replication.

In summary, for the first time we elucidated that in BHV-1 infected MDBK cells, all three major MAPK pathways are activated in response to viral infection, but JNK signaling was uniquely required for viral replication. Interestingly, we provide evidence that BHV-1 activated the MAPK pathways with a ROS-independent mechanism, which was different from that with HSV-1. The potential participation of these pathways in diverse processes of BHV-1 infection would provide valuable information towards comprehending the infection and inflammatory mechanism of BHV-1 infection in bovines.
